# Dual-Energy X-Ray Absorptiometry, Skinfold Thickness, and Waist Circumference for Assessing Body Composition in Ambulant and Non-Ambulant Wheelchair Games Players

**DOI:** 10.3389/fphys.2015.00356

**Published:** 2015-11-27

**Authors:** Annika Willems, Thomas A. W. Paulson, Mhairi Keil, Katherine Brooke-Wavell, Victoria L. Goosey-Tolfrey

**Affiliations:** ^1^The Peter Harrison Centre for Disability Sport, School of Sport, Exercise and Health Sciences, Loughborough UniversityLoughborough, UK; ^2^Lilleshall National Sport Centre, English Institute of SportSheffield, UK; ^3^School of Sport, Exercise and Health Sciences, Loughborough UniversityLoughborough, UK

**Keywords:** spinal cord injury, paralympic, total body mass, basketball, rugby

## Abstract

Field-based assessments provide a cost–effective and accessible alternative to dual-energy X-ray absorptiometry (DXA) for practitioners determining body composition in athletic populations. It remains unclear how the range of physical impairments classifiable in wheelchair sports may affect the utility of field-based body composition techniques. The present study assessed body composition using DXA in 14 wheelchair games players who were either wheelchair dependent (non-walkers; *n* = 7) or relied on a wheelchair for sports participation only (walkers; *n* = 7). Anthropometric measurements were used to predict body fat percentage with existing regression equations established for able-bodied persons by Sloan and Weir, Durnin and Womersley, Lean et al, Gallagher et al, and Pongchaiyakul et al. In addition, linear regression analysis was performed to calculate the association between body fat percentage and BMI, waist circumference, sum of 6 skinfold thickness and sum of 8 skinfold thickness. Results showed that non-walkers had significantly lower total lean tissue mass (46.2 ± 6.6 kg vs. 59.4 ± 8.2 kg, *P* = 0.006) and total body mass (65.8 ± 4.2 kg vs. 79.4 ± 14.9 kg; *P* = 0.05) than walkers. Body fat percentage calculated from most existing regression equations was significantly lower than that from DXA, by 2 to 9% in walkers and 8 to 14% in non-walkers. Of the anthropometric measurements, the sum of 8 skinfold thickness had the lowest standard error of estimation in predicting body fat content. In conclusion, existing anthropometric equations developed in able-bodied populations substantially underestimated body fat content in wheelchair athletes, particularly non-walkers. Impairment specific equations may be needed in wheelchair athletes.

## Introduction

Body composition measurement is vital in high performance sport because of the association of body fat and lean tissue mass with performance as well as health outcomes. Dual energy x-ray absorptiometry (DXA) is considered a relatively valid and reliable method to determine body composition in both able-bodied (Stewart and Sutton, 2012) and spinal cord injured (SCI) (Jones et al., 1998; Keil et al., 2014) individuals. However, the financial and logistical restrictions of DXA can limit accessibility for many practitioners and field-based body composition assessments are frequently employed as an alternative (e.g., waist circumference, skinfold thickness). Studies have shown body mass index (BMI), waist circumference and skinfold thickness to correlate well with total body fat and trunk fat and prediction equations have been developed to estimate body composition from these measurements (Eston et al., 2005; Weerarathna et al., 2008; Camhi et al., 2011). However, these relationships are population specific, differing according to gender and ethnicity due largely to different tissue distributions between these groups (Schreiner et al., 1996; Hill et al., 1999; Rahman et al., 2009; Camhi et al., 2011). Wheelchair game players present a range of lower and upper limb impairments that may also affect the relationships upon which such prediction equations are based. The accuracy of field-based methods for assessment of body composition in wheelchair game players is not known.

Lower limb paralysis and subsequent atrophy of lean mass following a SCI predisposes individuals to a decreased fat free mass (Maggioni et al., 2003; Stewart and Sutton, 2012), an increased fat mass (Spungen et al., 2003; Emmons et al., 2011) and a reduced resting metabolic rate (Buchholz and Pencharz, 2004). Previously, Spungen et al. (2003) observed comparable total lean and mass between individuals with thoracic and cervical level SCI. In contrast, some wheelchair games players with lower degrees of physical impairment remain ambulant in activities of daily life, only requiring a wheelchair for sports performance. The divergent fat distribution and lean mass profiles of individuals with varying degrees of physical impairments, and therefore modalities of daily ambulation, presents a challenge for cross-sectional and longitudinal body composition assessment. In addition, the standardized position to measure waist circumference and skinfolds in able-bodied persons is with the participant positioned in a standing anatomical reference position (Lohman et al., 1988). However, as this is not possible for most SCI-persons, waist circumference and skinfold thickness must be measured in supine or seated positions. These procedural differences might also affect the relationship between waist circumference, skinfold thickness and body fat and so affect the assessment of body composition by anthropometric techniques.

Two previous studies (Miyahara et al., 2008; Sutton et al., 2008) compared the body composition and fat distribution of male wheelchair athletes with able-bodied athletes and found a significantly lower lean body mass (Miyahara et al., 2008), higher body fat percentage (Miyahara et al., 2008; Sutton et al., 2008) and greater trunk fat mass (Sutton et al., 2008) in wheelchair athletes. In contrast, Sutton et al. (2009) did not find differences between the lean tissue mass and fat mass of the trunk when female SCI athletes and able-bodied non-athletic groups were compared. In addition, several studies (Bulbulian et al., 1987; Maggioni et al., 2003; Sutton et al., 2009) have investigated the applicability of using BMI, waist circumference, and skinfold thickness to estimate body fat percentage in wheelchair athletes using conventional regression-based body composition equations developed for able-bodied populations. These studies found that most regression equations (Sloan and Weir, 1970; Durnin and Womersley, 1974; Lean et al., 1996) underestimated body fat percentage for elite female wheelchair athletes (Sutton et al., 2009) and male paraplegic athletes (Bulbulian et al., 1987; Maggioni et al., 2003), with inaccuracy increasing with higher body fat percentages (Sutton et al., 2009). Interestingly, Sutton et al. (2009) suggested that regression equations that included the waist circumference appear to predict body fat percentage more accurately.

No distinction has yet been made between the body composition of athletes who are wheelchair dependent for daily activities and those that were ambulant yet eligible to compete in wheelchair sports using a sports wheelchair (Goosey-Tolfrey, 2010). The physical and disability characteristics are likely to influence the degree of relationship between field-based body composition assessment and DXA, as well as the accuracy of fat percentage prediction equations. The present study aimed to establish the accuracy of fat percentage prediction equations within wheelchair game players who were ambulant for daily living (walkers) and those who relied on wheelchair propulsion for daily ambulation (non-walkers). A secondary objective was to investigate the association between commonly employed field-based and DXA derived methods of determining body fat percentage in these two groups.

## Materials and methods

### Participants

Fourteen elite male wheelchair game players were recruited from national wheelchair basketball and national wheelchair rugby squads. All participants visited the laboratory once within the same competitive phase of their training schedule. The participants were divided into two groups; participants who were wheelchair independent during non-sporting activities (7 walkers) and daily wheelchair users (7 non-walkers). The participants' characteristics are shown in Table 1. The walkers comprised of five persons with single lower-limb amputations and two with lower limb deficiencies whilst the non-walkers comprised of all SCI-persons (motor complete SCI; C5–C7). All participants provided written informed consent prior to data collection and the study was approved by Loughborough University's Ethics Committee and the National Research Ethics Service.

**Table 1:** **Participant characteristics, DXA-derived body composition, BMI, waist circumference, and sums of 6 and 8 skinfold thickness**.

	**Walkers (*n* = 7)**	**Non–Walkers (*n* = 7)**	***P*-value (ES)**
Age (years)	26 ± 8	32 ± 7	0.15 (0.8)
Time since injury (years)	19 ± 10	12 ± 7	0.16 (0.8)
Sport	WCB	WCR	n/a
Physical impairment	Amputee (*n* = 5); Lower limb deficiency (*n* = 2)	SCI (*n* = 7)	n/a
Body mass (kg)	79.4 ± 14.9	65.8 ± 4.2	0.05^*^ (1.1)
Fat mass (kg)	16.9 ± 7.6	16.3 ± 5.3	0.88 (0.8)
Fat percentage (%)	21.4 ± 5.9	26.2 ± 8.9	0.25 (0.6)
Lean tissue mass (kg)	59.4 ± 8.2	46.2 ± 6.6	0.01^*^ (1.3)
Lean tissue percentage (%)	75.6 ± 5.5	70.2 ± 9.0	0.21 (0.7)
BMI	23 ± 4	21 ± 2	0.10 (0.9)
Waist circumference (cm)	85.5 ± 8.6	77.9 ± 7.8	0.11 (0.9)
Sum of 6 skinfold thicknesses (mm)	77.2 ± 18.6	85.0 ± 39.9	0.65 (0.3)
Sum of 8 skinfold thicknesses (mm)	102.2 ± 26.6	114.0 ± 47.0	0.57 (0.3)

All date are mean ± standard deviation. ES, Effect size; WCB, Wheelchair Basketball; WCR, Wheelchair Rugby; SCI, Spinal Cord Injury; BMI, Body Mass Index; Significant differences are indicated with

* *at a significant level of P < 0.05*.

### Anthropometry

The anthropometric measurements performed were: height, body mass, waist circumference, and skinfold thickness. Height was measured in a standing position (walkers) or a supine position (non-walkers), using a Luftkin measuring tape. Body mass was measured using a wheelchair accessible scale (Detecto 6550KGEU Portable, Detecto Scale Company, Webb City, Mo, USA). Participants who were not able to stand on the scale were weighed in their wheelchair, afterwards the wheelchair was weighed and its weight was subtracted from the total weight. Height (m) and body mass (kg) were used to calculate the BMI by the following formula:

$$\text{BMI}=\text{bodymass}/{\text{height}}^{2}$$

Waist circumference was measured directly to the skin using an inelastic tape at the narrowest part of the torso after normal expiration (Lohman et al., 1988; Buchholz and Bugaresti, 2005). For walkers, waist circumference was measured in a neutral standing position; for non-walkers, waist circumference was measured in a supine position with their arms at their sides. Waist circumference was measured three times and the average of these three measurements was taken for further analysis.

Skinfold thickness were measured at eight sites and were performed according to the guidelines from the ISAK (Lohman et al., 1988), having the same investigator measuring all skinfolds by using a set of Harpenden Skinfold Calipers (Baty International, West Sussex, UK). Skinfold sites included: biceps, triceps, subscapular, iliac crest, supraspinale, abdominal, anterior thigh, and medial calf. For the non-walkers, skinfolds were measured in a seated position, while the walkers were measured in a standing position. Each skinfold measurement was made in triplicate; true skinfold thickness was taken as the average of those three measures. However, in case of any outliers, the average of two measurements was taken. Afterwards, skinfold thickness were used to calculate the sum of 6 skinfold thicknesses (biceps, triceps, subscapular, iliac crest, supraspinale, and abdominal) and the sum of 8 skinfold thicknesses (biceps, triceps, subscapular, iliac crest, supraspinale, abdominal, thigh, and calf).

### Dual energy X-ray absorptiometry

Body composition was measured using DXA Lunar Prodigy Advance (GE Medical Systems, Madison, WI, USA) with Encore software version 13.2. The participants were instructed to wear loose fitting clothes without any metal and to remove all metal fixtures such as jewelry. Afterwards, the participants were positioned on the DXA-bed in a supine position as close as possible to standard positioning protocol, with Velcro straps used to help keep the legs still during measurement. Although some participants had muscular spasms during positioning, they were able to remain still once positioned. Scanning time varied between 6 and 10 min depending on the mass and height of the participants. The analyses of all scans were performed by the same operator to avoid any inter-observer variability. The total body scan was used to gain the outcome measures which were: total body fat mass, total body fat percentage, total lean tissue mass (with segmental data of the trunk, arms, and legs), lean tissue percentage and segmental fat mass of the trunk, arms, and legs.

### Fat percentage predicting regression equations

To investigate whether traditional fat percentage predicting regression equations were applicable to elite male wheelchair game players the data acquired from the anthropometry measurements were used to predict body fat. The predicted body fat was than compared to the data resulting from the DXA scan. The regression equations to which the data were applied are from Sloan and Weir (1970), Durnin and Womersley (1974), Lean et al. (1996), Gallagher et al. (2000), and Pongchaiyakul et al. (2005).

### Statistical analyses

All statistical tests were performed using IBM SPSS Statics 22.0 and statistical significance was set *a priori* at *p* < 0.05. Firstly, the assumption of normal distribution was checked for all data by visual inspection of the box plot and q-q plot and also the Shapiro-Wilk test within the groups. Equality of variance was checked using the Levene's test. As all data were normally distributed, independent *t*-tests were used to compare body mass, DXA derived fat mass, body fat percentage, lean tissue mass, lean tissue percentage, and waist circumference, BMI and sum of 6 skinfold thickness and sum of 8 skinfold thickness between the walking and non-walking group. Further, segmental lean and fat mass for the trunk, arms, and legs were compared between groups. For all variables the 95% CI for the mean difference was determined and effect sizes were calculated for all variables using the method of Cohen (1992), where an effect size of 0.2 represents a small effect, 0.5 a moderate effect, and 0.8 a large effect. To test the agreement between the results of the fat percentage predicting regression equations and DXA outcomes, the statistical method of Bland and Altman (2012) was used. Paired *t*-tests were performed for all five equations between the results of the DXA and equations to check for significant systematic errors. Also, Pearson's correlations were performed to check for proportional biases and heteroscedasticity. For the walkers and non-walkers, the association between body fat percentage and BMI, waist circumference, sum of 6 skinfold thicknesses and sum of 8 skinfold thicknesses was calculated by performing linear regression analysis and interpretation of slope and intercept.

## Results

### Body composition

**Figure 1:**
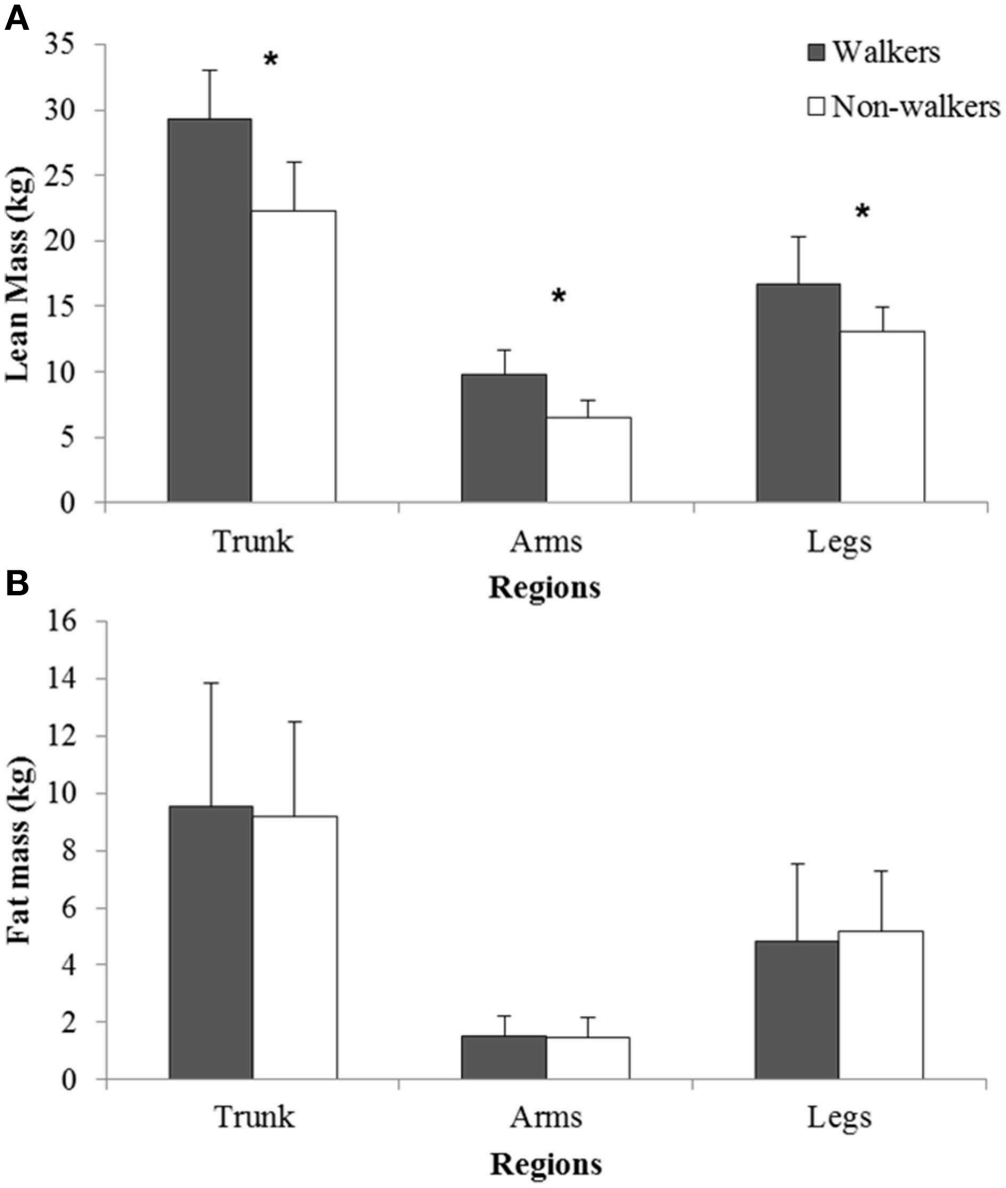
**Comparison of (A) segmental (trunks, arms, legs) lean tissue mass and (B) fat mass for walkers and non-walkers**. ^*^ Significant difference between groups (*P* < 0.04).

There were no significant differences for age and time since injury between the two groups (Table 1). Table 1 shows the physical characteristics of the two groups, where non-walkers were lighter than walkers (*P* = 0.05) with significantly lower lean tissue mass than the walking group (*P* < 0.01). Lean tissue mass was significantly lower (*P* < 0.04; effect size > 1.0) across the trunk, arms, and legs for non-walkers (Figure 1). For the other variables (fat mass, fat percentage, lean tissue percentage, segmental fat mass, BMI, waist circumference, sum of 6 skinfold thicknesses, and sum of 8 skinfold thicknesses) no significant differences between the two groups were found. However, the effect sizes of BMI and waist circumference were 0.9, indicating substantially, but not significantly, lower BMI and waist circumference in the non-walkers.

### Fat percentage prediction equations

**Figure 2:**
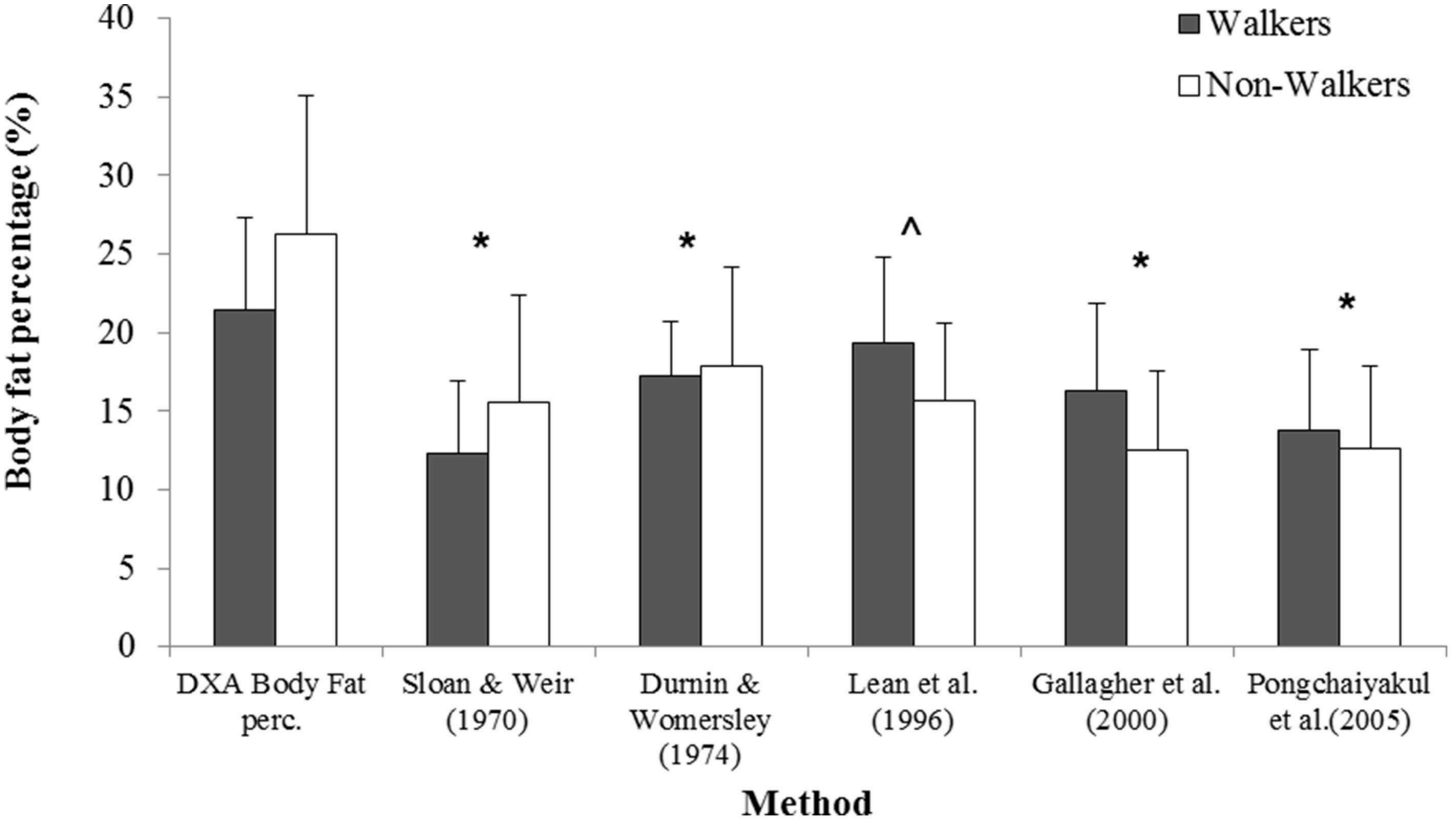
**Body fat percentage derived by DXA scan and the fat percentages calculated by the anthropometric prediction equations for walkers and non-walkers**. ^*^ Both groups significantly lower than DXA (*P* < 0.05), ^ Non-walkers significantly lower than DXA (*P* < 0.05).

The results of body fat percentage derived by DXA scan and the fat percentages calculated by the anthropometric prediction equations for walkers and non-walkers can be found in Figure 2. The agreement between the percent fat estimated from DXA and anthropometric predictions equations, displayed as mean percentage systematic error and 95% limits of agreement, are shown in Table 2. Pearson's correlations revealed that there were no proportional biases or heteroscedasticity within the walking group (*P*≥0.93) and non-walking group (*P*≥0.142). In the walking group, the equation of Lean et al. (1996) did not show a significant difference between the predicted body fat percentage and percent body fat measured by DXA. Also, this equation showed the lowest systematic error which was an underestimation of 2.1% of body fat percentage. The limits of agreements showed that the equation of Lean et al. (1996) will, 95% of the time, produce body fat percentage estimates between 9.7% less and 5.5% more than the DXA value. All other formulae showed significant differences between the equation outcomes and DXA measured body fat percentage (*P* ≤ 0.04). For the non-walking group (see Table 2), all formulae showed significant differences between the equation outcomes and DXA measured body fat percentage, with underestimated body fat percentage and a large systematic errors (ranging from 8.3 to 13.7%).

**Table 2:** **Agreement between DXA-determined percent body fat and values given by anthropometric equations for walkers (*n* = 7) and non-walkers (*n* = 7)**.

		**Mean bias in % fat (anthropometry- DXA; mean ± SD)**	***p*-value for bias**	**95% limits of agreement (Bland and Altman)**		
				**Lower**	**Upper**	**Range**
Walkers	Sloan and Weir, 1970	−9.0±2.6%	< 0.01^*^	−14.0	−4.0	10.0
	Durnin and Womersley, 1974	−4.2±3.8%	0.03^*^	−11.6	+3.3	14.9
	Lean et al., 1996	−2.1±3.9%	0.21	−9.7	+5.5	15.3
	Gallagher et al., 2000	−5.1±5.3%	0.04^*^	−15.5	+5.3	20.8
	Pongchaiyakul et al., 2005	−7.7±5.0%	< 0.01^*^	−17.5	+2.1	19.6
Non-walkers	Sloan and Weir, 1970	−10.6±4.5%	< 0.01^*^	−19.4	−1.78	17.6
	Durnin and Womersley, 1974	−8.3±4.8%	< 0.01^*^	−17.7	+1.1	18.8
	Lean et al., 1996	−10.6±7.4%	< 0.01^*^	−25.1	+3.9	29.0
	Gallagher et al., 2000	−13.7±7.5%	< 0.01^*^	−28.4	+1.0	29.4
	Pongchaiyakul et al., 2005	−13.6±6.5%	< 0.01^*^	−26.3	−0.9	25.5

Eq., Equations; SD, Standard Deviation. Significant differences are indicated with

* *at a significant level of P < 0.05*.

### Regression analysis

Waist circumference was associated with percentage fat in walkers only (Table 3). However, the relationship appeared different between the groups, particularly for waist circumference (Table 3). This is highlighted by the lower percentage fat, but higher waist circumference, in walkers than non-walkers (Table 1). BMI was not significantly associated with percentage fat in either group (Table 3) and the standard error of estimate of percentage fat from BMI was large, particularly in the non-walkers. The sums of 6 or 8 skinfold thickness were significantly associated with % fat in both groups, with standard errors of estimate within 5% in both groups. Also, the relationship between the sum of skinfold thicknesses and % fat did not seem to differ so substantially between groups (Table 3).

**Table 3:** **Linear regression analysis of anthropometric measures and DXA-derived percentage body fat**.

	**Correlation co-efficient (*****r*****)**		**SEE**		**Intercept**		**Slope (95% CI)**	
	**Walkers**	**Non-Walkers**	**Walkers**	**Non-Walkers**	**Walkers**	**Non-Walkers**	**Walkers**	**Non-Walkers**
WC	0.79^*^	0.62	4.00	7.61	−24.7	−29.37	0.54 (0.05 to 1.21)	0.71 (−0.32 to 1.74)
BMI	0.49	0.59	5.65	7.83	2.5	−21.35	0.81 (−0.84 to 2.46)	2.32 (−1.2 to 5.93)
Sum of 6	0.84^*^	0.87^*^	3.54	4.78	0.81	9.75	0.27 (0.07 to 0.47)	0.19 (0.07 to 0.32)
Sum of 8	0.98^*^	0.88^*^	1.16	4.65	−0.95	7.32	0.22 (0.17 to 0.26)	0.17 (0.06 to 0.27)

WC, waist circumference; BMI, body mass index; SEE, standard error of the estimate. Significant correlations are indicated with

* *at a significant level of P < 0.05*.

## Discussion

The estimation of body fat percentage in wheelchair game players is a difficult task due to the variety of disabilities and the consequent differences in distribution of body tissues from able-bodied persons. This may explain the paucity of studies that have evaluated the use of predictive equation techniques from anthropometric measurements in this population. The primary finding of our study is that fat percentage predicting regression equations, based on skinfold thickness, waist circumference, age, and sex, used in the able-bodied populations are not applicable to ambulant and non-ambulant wheelchair sportspersons. Due the strong correlations and positive association found for sum of 6 skinfold thicknesses and sum of 8 skinfold thicknesses in non-walkers and walkers with body fat percentage, respectively, these variables could be useful to establish regression equations in the future for non-walking and walking wheelchair game players.

The results showed a difference in body mass between the groups, caused by a significantly lower lean tissue mass in the non-walkers as the fat mass was not significantly different between the groups. Segmental data for the trunk, arms, and legs showed lower lean tissue across all regions, representing the muscular atrophy associated with paralysis and impaired muscle innervation. These findings are in is line with findings of other studies who found significantly lower lean tissue mass in SCI-individuals (Maggioni et al., 2003; Miyahara et al., 2008; Stewart and Sutton, 2012). Previous studies showed a significantly higher body fat percentage for wheelchair athletes compared to able-bodied controls (Miyahara et al., 2008; Sutton et al., 2008). It could have been expected that this increased fat percentage in wheelchair athletes is caused by the increased fat mass of the SCI-athletes, as people with SCI are previously reported to have a higher fat mass (Spungen et al., 2003; Emmons et al., 2011). However, our results showed that DXA derived body fat mass in the walkers is not significantly different form the non-walkers. The main difference in body composition between non-walkers and walkers is thus lean tissue mass and not a difference in fat mass. BMI and waist circumference showed no significant differences between the groups but the effect sizes were very high.

The equations that were applied in this study were all established for the able-bodied population. The results showed that they are not transferrable to the non-walking or the walking athletic wheelchair population. For the walkers, the equation of Lean et al. (1996) predicted a fat percentage that was not significantly different from DXA-measured fat percentage. However, the limits of agreement were wide, making the prediction very inaccurate. Therefore, it is not advisable to use the equation for ambulant disabled wheelchair game players. All other equation based predictions for the non-walkers and walkers were significantly different from DXA-measured fat percentage, and are thus not usable to estimate body fat percentage in these populations. Our results are in agreement with the findings from Sutton et al. (2009), Maggioni et al. (2003), and Mojtahedi et al. (2009) who also concluded that the equations underestimate body fat percentage and are not accurate enough to use in daily practice.

No significant correlations between BMI and body fat percentage were found for either the walkers or non-walkers, in contrast to the results of Sutton et al. (2009) who found a strong correlation in female wheelchair athletes. The large standard error of estimation of percentage fat from BMI demonstrates that BMI is a particularly poor indicator of obesity in wheelchair athletes. Waist circumference correlated with body fat percentage in the walkers which is in agreement with the work of Sutton et al. (2009), however, no significant correlation was not found for the non-walkers. Despite the more centrally located fat mass in SCI (Spungen et al., 2003), non-walkers tended to have lower waist circumference at any given fat percentage than walkers, although the sample size was not sufficient to detect a significantly different relationship between the two groups. In contrast to Sutton et al. (2009) suggestion, the high SEE of fat percentage estimated from waist circumference suggests that waist circumference does not provide a better indicator of body fat content than skinfolds in this population.

The sums of 6 and 8 skinfold thickness showed the highest correlation with body fat content in this sample. A closer look at those correlations shows that for non-walkers, the correlation of sum of 6 skinfold thickness and sum of 8 skinfold thickness are similar, although the lower standard error of estimate with 8 skinfold thickness suggests this measure may provide most accuracy. Although existing skinfold thickness equations developed in able-bodied populations substantially underestimated body fat content, in this sample, the relatively low SEE for the regression of fat percentage and skinfold thickness suggests that it may be possible to derive population specific equations for wheelchair athletes based on skinfold thickness. Theoretically, it may be desirable to also include a variable that may reflect the atrophy of lean tissue, e.g., thigh circumference. Furthermore, given that height may be substantially affected in double amputees and also some SCI, an alternative index of body size such as arm-span may be more appropriate in this population. However, one difficulty in developing such equations is the limited sample of wheelchair athletes, and a further difficulty is the heterogeneity of the population. Pending any future developments in body composition assessment in these groups, we suggest that body composition be assessed by DXA, and any estimates from anthropometric equations be adjusted accordingly.

In the present study DXA was used to assess body composition and not a four component model, which is standard better criterion measure for assessing body composition. Although some studies found very high precision of DXA in able-bodied persons (Stewart and Sutton, 2012) and in SCI individuals (Jones et al., 1998), the accuracy of DXA for assessing body composition is still debated (Van Loan and Mayclin, 1992; Glickman et al., 2004). Therefore, inaccuracies in body composition measurements might have occurred in this study. However, considering the reported accuracy of DXA (Jones et al., 1998; Stewart and Sutton, 2012), possible inaccuracies in assessing body composition should be relatively small. Another limitation of this study might be the small sample size. As the study deals with a highly specific population (Paralympic wheelchair athletes) it is hard to increase the sample size number. Despite the low participant number we were still able to uncover interesting results about the body composition of athletes classified as non-walkers and walkers. This study is the first study trying to assess body composition in wheelchair athletes with anthropometric measurements, differentiating between non-walkers and walkers. Given the interesting results of this study, further research into body composition assessment of non-walking and walking athletes should be able to establishing separate field-methods for body composition assessment within these populations.

## Conclusion

In conclusion, there were notable differences for the total lean tissue mass between the non-walkers and walkers. Non-walkers displayed significantly lower segmental lean mass of the trunk, arms, and legs, resulting in significant differences in total body mass between groups. The regression equations developed from able-bodied populations using BMI, waist circumference, age, and skinfold thickness measurements substantially underestimated body fat content, particularly in non-walkers. All formulae, apart from that of Lean et al.'s (1996) in the walkers, showed significant differences between the equation outcomes and DXA-derived body fat percentage, with an underestimation in body fat percentage and large systematic errors across present in all equations (ranging from 8 to 14%). Due the strong correlation between sum of 8 skinfold thickness and body fat percentage, and the low SEE for walkers and non-walkers, this variable could be suitable to establish future impairment specific regression equations for wheelchair game players.

### Conflict of interest statement

The authors declare that the research was conducted in the absence of any commercial or financial relationships that could be construed as a potential conflict of interest.
